# Optical Sensors Based on Whispering Gallery Modes in Fluorescent Microbeads: Size Dependence and Influence of Substrate

**DOI:** 10.3390/s90906836

**Published:** 2009-08-31

**Authors:** Alexandre Francois, Michael Himmelhaus

**Affiliations:** 1 Fujirebio, Inc. / 51 Komiya-cho, Hachioji-shi 192-0031, Tokyo, Japan; 2 Present address: Centre of Expertise in Photonics / School of Chemistry and Physics, The University of Adelaide, 5005 Australia; E-Mail: alexandre.francois@adelaide.edu.au (A.F.)

**Keywords:** whispering gallery modes, cavity modes, optical sensing, refractive index sensing, biosensing

## Abstract

Whispering gallery modes in surface-fixated fluorescent polystyrene microbeads are studied in view of their capability of sensing changes in the refractive index of the beads’ environment by exposing them to water/glycerol mixtures of varying composition. The mode positions are analyzed by simultaneous fitting for mode number, bead radius, and environmental index. Down to a diameter of 8 μm, the sensor response follows the index of the bulk solution very well. For smaller bead sizes, some deviations occur, in particular for fluid indices not too different from that of water, which might be attributed to the presence of the substrate.

## 1. Introduction

Optical sensors based on whispering gallery mode (WGM) excitations in fluorescent microbeads have recently been introduced for remote refractive index sensing [1,2] and biosensing [3,4] with an aim to establish a novel class of highly sensitive, remotely operable optical microsensors. In contrast to most evanescent field sensors, such as fiber sensors [5,6], optical waveguides [7,8], and surface plasmon resonance [9,10], which apply freely traveling waves, WGM are optical cavity mode excitations obeying a closed resonator condition, which renders them sensitive to the microcavity’s geometry [11,12]. Because of this peculiarity, WGM sensors promise to achieve improved sensitivity and performance compared to state-of-the-art evanescent field sensors, in particular on the micro-scale, where the sensor’s size may be subject to non-negligible changes in the course of (bio-) molecular interactions.

Because of their small dimension, i.e., small radius R, microbeads exhibit a wide free spectral range, *δλ ∝ λ^2^/R*, of several nanometers when operated as microcavities. Therefore, the cavity mode spectrum of a microbead can be easily exploited over a wide spectral range by means of a simple spectroscopic system, thereby yielding a wealth of information on the system in terms of mode positions and bandwidths. This is in contrast to the well-established sub-millimeter cavities that have found various applications as optical sensors [13,14] and biosensors [15], which however—due to their extremely narrow free spectral range—typically apply single mode tracking by means of an ultra narrowband tunable light source.

Fluorescence excitation has proven to be a very convenient and versatile way of WGM analysis over a wide spectral range [16] and thus promises to improve WGM sensor performance due to the high information content obtainable. This is important because typically a number of parameters, such as the microbead’s size and the refractive index of the bead’s environment are not exactly known at the beginning of a sensing process. Recently, Zijlstra *et al*. demonstrated that for remote index sensing, the exact size of the microbead does not need to be known as long as the size dispersion of the microbead suspension is sufficiently small [1]. Further, the authors showed that as long as the sensor surface is sufficiently clean, the refractive index could be calculated from mode spacing and bandwidth. For biosensing, however, such simplified approach seems not to be suitable for a number of reasons. First of all, in contrast to index sensing, in biosensing an additional layer is formed on the sensor surface, thereby complicating data analysis by introducing additional parameters as well as by jeopardizing the “clean surface” requirement. Most crucially, as Arnold and coworkers [12] have pointed out, the WGM shift in a microsphere of radius R induced by this adsorption layer is proportional to 1/R, thus demanding for precise determination of the initial sensor radius. In a colloidal suspension of fluorescent microbeads, however, the latter cannot always be assessed in a reference experiment, therefore requiring a more sophisticated data evaluation than those used for index sensing [1,2]. Also, the sensors are typically surface-attached to allow multiple process steps in a bio-recognition experiment or to facilitate multiple analyte detection. Finally, from a practical point of view, application of colloidal suspensions with very narrow size dispersion seems not to be feasible in terms of costs and efforts.

Therefore, in the present article we explore the potential of a more rigorous data analysis in view of simultaneous determination of all relevant parameters, such as mode assignments, bead radius and refractive index of its ambient, from the measured WGM positions. By exposing sensors of different sizes to fluids of varying refractive indices, the accuracy of this evaluation can be directly assessed in dependence of all of these parameters. This is particularly important for in-situ biosensing because of the 1/R dependence of the WGM shift, which suggests a minimization of sensor dimension for accomplishment of ultimate sensitivity and thus demands for its thorough determination.

## 2. WGM Simulation

For the theoretical description of the WGM positions, we apply the Airy approximation [17] for microspheres in a dielectric medium as recently given by Pang *et al*. for transverse electric (TE) and transverse magnetic (TM) modes [2]:

(1a) $$\lambda^{\mathrm{TE}}(q=1,\;\ell ,R,m)=2\;\pi \;n_{s}\;R\;{\left(\nu +1.8557\;\nu^{1/3}-\frac{m}{\sqrt{m^{2}-1}}+1.0331\;\nu^{-1/3}-\frac{0.6186\;m^{3}}{{\left(m^{2}-1\right)}^{3/2}}\;\nu^{-2/3}+O\left(\nu^{-1}\right)\right)}^{-1}$$

(1b) $$\lambda^{\mathrm{TM}}(q=1,\ell ,R,m)=2\;\pi \;n_{s}\;R\;{\left(\nu +1.8557\;\nu^{1/3}-\frac{1}{m\;\sqrt{m^{2}-1}}+1.0331\;\nu^{-1/3}-\frac{1.8557\;\left(m^{4}-\frac{2}{3}\right)}{m^{3}\;{\left(m^{2}-1\right)}^{3/2}}\;\nu^{-2/3}+O\left(\nu^{-1}\right)\right)}^{-1}$$

Here, *λ^TE^* and *λ^TM^* describe the wavelength positions of first order, i.e., q = 1, TE and TM modes with mode number ℓ, n_s_ represents the microbead’s refractive index, R its radius, m = n_s_/n_e_ the refractive index contrast at the bead/environment interface, where n_e_ is the refractive index of the environment, and $\nu =\ell +\frac{1}{2}$. For further details of the mode assignment, we refer to the literature [11].

The advantage of using these approximations, which deviate from the exact solutions only by an error of the order of ν^−1^, is simply that equations 1 comprise analytical functions that can be easily implemented into a fitting routine for simultaneous determination of the parameters ν, m, and R, while calculation of the exact solutions involves a tedious numerical procedure, incl. the multiple use of Bessel functions, whose application in a fitting algorithm is presently not feasible on a personal computer.

**Figure 1.**
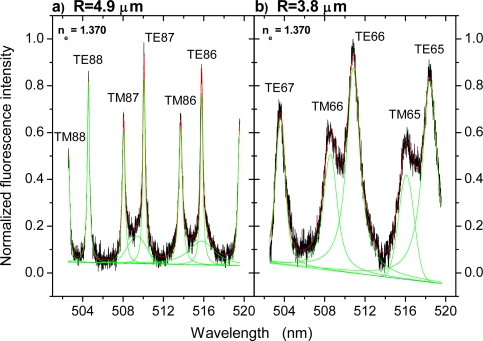
Illustration of the fitting of Voigt profiles and linear background correction to the measured WGM spectra for determination of mode positions and bandwidths. (a) Spectrum of the R = 4.9 μm bead at a fluid index, n_fl_ = 1.370. (b) Spectrum of the R = 3.8 μm bead at the same fluid index. The mode assignments (polarization followed by mode number, ℓ) are based on the results of the spectrum evaluation.

For determination of the parameters, the spectra obtained were first fitted by means of Voigt profiles applying either linear or 4^th^ order background correction. We used Voigt profiles instead of Lorentzians to account for a potentially present small inhomogeneous broadening imposed by small deviations of the beads’ shape from sphericity [18]. In particular for larger beads with sizes of about 10 μm it is important to fit all modes simultaneously including proper background correction, because some higher order modes with bandwidths of several nanometers [1] contribute to the background and have to be accounted for by use of additional Voigt profiles and occasionally by applying a non-linear background correction. Two examples of the peak fitting procedure are given in Figure 1 for illustration.

With the measured mode positions, ${\bar{\lambda }}_{i}^{\mathrm{TM}}$ and ${\bar{\lambda }}_{i}^{\mathrm{TE}}$, precisely determined, the free parameters of Equation 1, which are ν (or alternatively, ℓ), m (or alternatively, n_e_), and R, can be fitted by minimizing the deviation between measured and calculated mode positions:

(2) $$\Delta =\sum_{i,j}\left|{\bar{\lambda }}_{i}^{\mathrm{TM}}-\lambda_{i}^{\mathrm{TM}}(q=1,\ell_{i},R,m)\right|+\left|{\bar{\lambda }}_{j}^{\mathrm{TM}}-\lambda_{j}^{\mathrm{TM}}(q=1,\ell_{j},R,m)\right|$$

The only ambiguity in applying Equation 2 is related to the classification of the measured modes into TM and TE modes. This issue, however, can be easily resolved by applying Equation 1 to some approximate values for the parameters ν, m, and R, which then shows that for polystyrene beads of few micrometers in diameter in an aqueous ambient, TM and TE modes of same mode number ℓ show up in the spectra as well-separated pairs with ${\bar{\lambda }}_{\ell }^{\mathrm{TM}}<{\bar{\lambda }}_{\ell }^{\mathrm{TE}}$, thus allowing an assignment by eye (*cf.*, e.g., mode assignments in Figure 1). An initially chosen wrong assignment would further lead to an unsatisfying residual deviation Δ within the relevant parameter range.

On this basis, the free parameters were determined from the experimental WGM spectra at a precision of three digits for bead radii and four digits for refractive indices. Mode numbers were obviously determined as integers.

## 3. Results and Discussion

**Figure 2.**
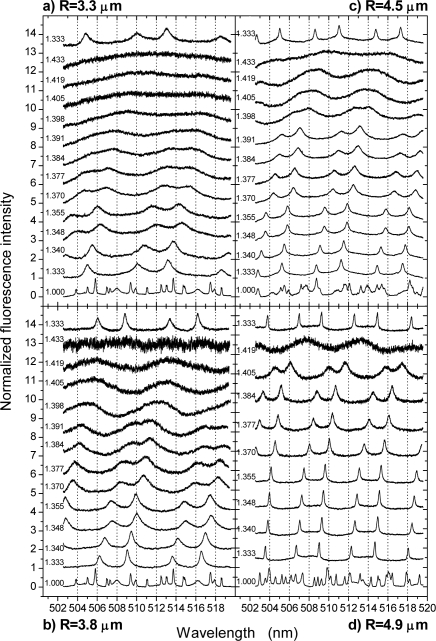
WGM spectra of four different beads of differing radii in dependence of the fluid index, n_fl_, of the DI water/glycerol mixture injected into the microfluidic flow cell. The spectra are labeled with their respective fluid indices as calculated from [19] and are vertically displaced for clarity.

Figure 2 displays four series of WGM spectra obtained from four fluorescent microsensors of different radii, R, exposed to fluids of different refractive indices, n_fl_. In each case, a spectrum in air was acquired first to judge the quality of the microresonator, then DI water/glycerol mixtures were subsequently injected into the microfluidic channel starting with pure DI water and then increasing the glycerol content up to 70%. Beyond 70% the fluid became too viscous for injection, which, however, did not matter much because the WGM spectra were fading out under the correspondingly high refractive indices anyway. Each experimental series was terminated with the acquisition of a WGM spectrum in water to assure that the entire procedure had no permanent effect on the respective microsensor.

The spectra taken in air exhibit a large number of modes, which can be assigned to first order TM and TE modes as well as higher order contributions. For details we refer to the literature [3,11]. It can be nicely observed that the number of modes per spectrum is decreasing with decreasing dimension of the microbeads, thus illustrating the aforementioned relation between bead radius and free spectral range.

When immersed into DI water, the spectra undergo a significant change. Because of the reduced index contrast at the interface, m = n_s_/n_e_, higher order modes have become too lossy with correspondingly low quality factors (Q-factors), Q = λ/Δλ, and broad bandwidths, Δλ, so that only first order modes remain clearly discernible. However, as illustrated in Figure 1a, for the largest beads under study some additional modes still need to be taken into account for spectrum fitting, though hardly discernible by eye. For spectra obtained from smaller microbeads, such as that one shown in Figure 1b, such additional modes are not required and the spectra were fitted by assigning peaks solely to the clearly discernible first order modes. Further, as can be seen from the mode assignment given in Figure 1, the TM/TE modes of given mode number ℓ do now show up as well separated pairs, whereby ${\bar{\lambda }}_{\ell }^{\mathrm{TM}}<{\bar{\lambda }}_{\ell }^{\mathrm{TE}}$. As evident from Figure 2, this behavior is the same for all bead radii and fluid indices throughout the entire parameter range studied.

With increasing fluid index, the interfacial refractive index contrast further reduces, thereby more and more also affecting the first order modes. This can be seen not only from their increasing red-shift, but especially from their increasing bandwidths, which reflect the increasing losses. Obviously, the smaller the microbead, the earlier the modes become too broad to remain unambiguously discernible.

To gain better understanding of these effects and to draw conclusions about the parameter range practically suitable for sensing in terms of minimum sensor radius, R, and maximum fluid index, n_fl_, the spectra shown in Figure 2 were analyzed in detail as described in the methods section. In short, the mode positions and bandwidths were determined by fitting of Voigt profiles as discussed above and illustrated in Figure 1. Then, the mode positions were used for simultaneous fitting of all relevant parameters according to Equations 1 and 2. From thus obtained parameters, in particular the calculated environmental indices, n_e_, experienced by the microsensors, conclusions about the precision of this method could be immediately drawn by comparison with the nominal fluid indices as determined by SPR.

**Figure 3.**
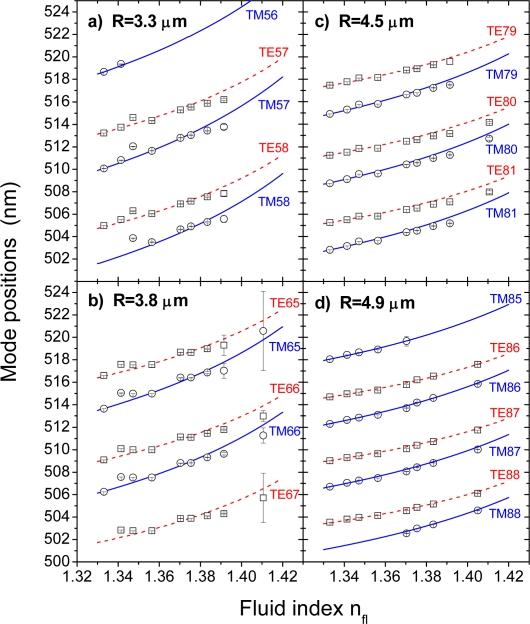
Wavelength positions of the WGMs of the four different beads shown in Figure 2 in dependence of the fluid index, n_fl_, of the DI water/glycerol mixture injected into the microfluidic flow cell. The solid (TM) and dashed lines (TE) were calculated using the Airy approximations given in Equation 1 and the best-fit parameters for ν, n_s_, and R resulting from an evaluation of the respective spectra obtained in DI water by assuming n_e_ = 1.333. The different modes are labeled according to their polarization, TM or TE, and mode number, $\ell =\nu -\frac{1}{2}$. The errors shown are the variances calculated by the least mean square fitting routine.

**Figure 4.**
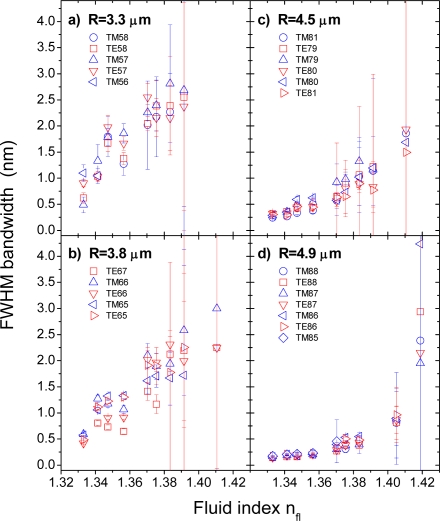
Full-width-half-maximum (FWHM) bandwidths of the WGMs of the four different beads shown in Figure 2 in dependence of the fluid index, n_fl_, of the DI water/glycerol mixture injected into the microfluidic flow cell. The different modes are labeled according to their polarization, TM or TE, and mode number, $\ell =\nu -\frac{1}{2}$. The errors shown are the variances calculated by the least mean square fitting routine.

Figures 3 and 4 display mode positions and bandwidths, respectively, as obtained from the spectrum evaluation as a function of the fluid index. The different modes have been assigned according to the results of the subsequent parameter fitting. Obviously, the mode positions red-shift with increasing fluid index, and the separation between TM and TE modes of same mode number reduces. The solid (TM modes) and dashed (TE modes) lines shown in Figure 3 are calculated according to Equation 1 by using the best-fit parameters for ν, m, and R as obtained from the fits to the respective DI water spectra (2^nd^ spectra from bottom in Figure 2). The good agreement between measured and calculated modes gives confidence for the validity of the Airy approximations (Equation 1) in the present parameter range and also allows us to determine a sensitivity limit of the sensors within the studied size regime. From linear fits to the four modes of the R = 3.3 μm bead shown in Figure 3a in the regime from n_e_ = 1.37 – 1.39, which is particularly interesting for biological applications, such as in-vitro cell studies [20], we obtain a detection limit of Δn_e_ = ±(2.1 ± 0.26) 10^−4^ as an average over all four modes and assuming a wavelength resolution of Δλ = ±0.01 nm. The latter value is based on our observations of the long-term stability of the fluorescent beads rather than the resolution of the detection system, which could be pushed further down, but we want to provide a practical and reasonable assessment here.

The evolution of the mode bandwidths with increasing fluid index as shown in Figure 4 demonstrate very nicely the effect of the decreasing index contrast at the microsensor/ambient interface. Further, they reveal the size-dependent losses, which are mainly due to the different curvature of microbeads of different radii, which affects the condition of total internal reflection (TIR) for the recirculating light. For large beads, i.e., R > 100 μm, the curvature is basically negligible and the light experiences TIR similar to that known for plane interfaces. With decreasing radius, however, the curvature affects more and more the TIR condition, thereby causing intrinsic size dependent losses. For the present parameter range in terms of bead radii, R, and refractive index contrasts, m, the size-dependent losses increase significantly with decreasing radii as can be seen from the bandwidths for DI water spectra shown to the most left in the four graphs of Figure 4 from about 0.2 nm at R = 4.9 μm, 0.3 nm at R = 4.5 μm, 0.5 nm at R = 3.8 μm to ∼0.7 nm at R = 3.3 μm.

To these intrinsic losses add those imposed by the decreasing index contrast when the fluid index is increased. For the largest bead shown in Figure 4, the error bars seem to be reasonably small up to a fluid index of about 1.39, beyond which any evaluation of the modes in terms of their bandwidths as proposed by Zijlstra *et al*. [1] becomes meaningless. With decreasing dimension, this limit can be found for ever smaller fluid indices.

To find out whether a more rigorous theoretical treatment can widen the suitable parameter range despite of the increasing errors in the bandwidths, the mode positions as shown in Figure 3 were used for simultaneous fitting of the free parameters ν, m, and R according to Equations 1 and 2. Since only the mode positions, which exhibit significantly smaller errors according to Figure 3, enter the equations, there is some hope that this procedure will in fact yield more precise results even for the limiting cases of small sensor dimension and high fluid indices, which is particularly important for biosensing due to the expected gain in sensitivity with decreasing sensor radius.

Only the refractive index contrast m = n_s_/n_e_ enters into Equation 1, not the absolute index values. Therefore, a precise value for the bead index n_s_ is required to assure proper determination of n_e_. According values for n_s_ were obtained for each bead individually by using the spectra obtained in DI water for simultaneous fitting of ν, n_s_, and R, thereby fixing the environmental index to n_e_ = 1.333 for DI water. In all subsequent fits of spectra obtained at higher fluid indices, n_s_ was kept constant at the value found for the respective microbead, thereby yielding values for ν, n_e_, and R. The values obtained for n_s_ are listed in Table 1. Given that they were independently determined from each other, they match surprisingly well, thus corroborating the validity of this procedure. Further, we found a systematic offset towards higher environmental indices, n_e_, when using the literature value of polystyrene beads of n_s_ = 1.590 [21].

**Figure 5.**
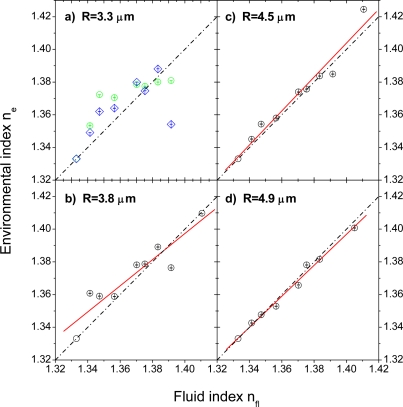
Environmental refractive index, n_e_, experienced by the different beads as determined by the fitting procedure in dependence of the fluid index, n_fl_, of the DI water/glycerol mixture injected into the microfluidic flow cell. The dash-dotted lines indicate the expected 1:1 behavior; the red solid lines are linear fits to the data. The errors shown are the Δ_±10%_ errors calculated from a variation of Equation 2. For details, see main text.

Figure 5 displays the calculated environmental refractive indices, n_e_, in dependence of the fluid index, n_fl_. Since a 1:1 behavior is expected, the n_e_ = n_fl_ relations are plotted as dash-dotted lines in the graphs as a guide to the eye. Further, except for the smallest bead radius, a linear fit to the respective data is plotted as solid line. Obviously, for the two largest bead radii of R = 4.9 μm and R = 4.5 μm, the environmental index, n_e_, follows the fluid index, n_fl_, very nicely. With decreasing bead radius, however, there is a deviation from this behavior, in particular for the smallest sphere of R = 3.3 μm. To confirm that this is not only an artifact of a single bead, the data of two beads of about same diameter are shown in Figure 5a (green and blue symbols, respectively). In both cases, initially, n_e_ rises with increasing n_fl_ more than expected but then exhibits a kind of saturation, which actually roughly brings the data back to the expected 1:1 behavior. It is not exactly clear why this initial strong increase in n_e_ happens, but it is—to lesser extent—also observable for the beads with radii of R = 3.8 μm and R = 4.5 μm, so that it seems to be connected to the sensor dimension. Potential causes could be related to a decreasing precision of the evaluation procedure with decreasing particle size, the presence of the surface, which might be better sensed by smaller beads, and finally, surface-related effects due to particular flow conditions or surface-fluid interactions in direct vicinity of the channel surface. These issues will be discussed in more detail in the following sections.

## 4. Discussion

The present study reveals that the environmental refractive index, n_e_, traced by surface-adsorbed and PE-coated fluorescent PS microbeads matches that of the fluid introduced into the microfluidic system very well down to a sensor diameter of about 9 μm. For a sensor size of 7.6 μm and below, some deviations from the expected behavior are observed, so that the present limit in size seems to be somewhere around 8 μm diameter. In the following, we will discuss the different potential causes for the deviation at smaller sizes in more detail to gain further insight into WGM sensing in particular in view of their importance for biosensing applications, which would benefit from a minimization of sensor dimension due to the 1/R dependence of the WGM shift upon molecular adsorption [12].

The first interesting question is whether above determined bead refractive index of about n_s_ = 1.56 reflects really the physical condition of the outer bead volume or whether it functions simply as a free parameter that tunes the results into the desired range. This could happen in particular because of the presence of the PE coating, which has a lower refractive index than PS, n_PE_ = 1.47 [22], and thus may contribute to an average index as experienced by the WGMs. The same arguments hold for the presence of the surface, which also has a lower index than PS (n_glass_ = 1.5255, as provided by the manufacturer, *cf.* e.g., www.matsunami-glass.co.jp).

To rule out the influence of both PE coating and surface on the result obtained for the bead index, we acquired WGM spectra from freely floating and uncoated commercial yellow-green fluorescent PS beads with a nominal diameter of 10 μm. WGM spectra of these beads were obtained immediately after placing a small droplet of highly diluted particle suspension onto a microscope cover slip, and then focusing onto microbeads before they settled on the surface. The height of the beads above surface was several tens of micrometers as could be concluded from the z-axis movement needed for focusing. Thus obtained spectra were evaluated in the same way as those before and n_s_ calculated by setting n_e_ = 1.333, which gave a value of n_s_ = 1.5638 ± 0.0047 in reasonable agreement with the results for the surface-adsorbed beads, which gave n_s_ = 1.5584 ± 0.0042 (*cf.* Table 1). It should be noted, however, that the surface-adsorbed beads studied are smaller in size on average and that there is a certain trend of decreasing index, n_s_, with decreasing radius observable (*cf.* Table 1). In fact, the agreement between the average index obtained with the freely floating beads matches perfectly that obtained for the largest surface-adsorbed bead with R = 4.9 μm. From our experience, we know that smaller beads are more susceptible to the doping procedure, which might be reflected by the trend of decreasing bead index observed here. Therefore, the data seem reliable and in conclusion, an influence of both PE coating and surface on the bead index can be excluded.

The validity of thus determined bead indices, n_s_, is further confirmed by the good match between the observed shifts in the mode positions and their theoretical curves based on Equation 1, both shown in Figure 3. The latter were calculated by varying only the environmental index, n_e_, while fixing n_s_ and R to their respective values as obtained from the DI water spectra. The excellent agreement also for higher environmental indices, n_e_, does not only corroborate the validity of the Airy approximations in the given size regime, but also the use of above determined bead indices, n_s_. Therefore, once more we conclude that bead indices given in Table 1 reflect the physical condition of the outer bead volume as it might be affected by the doping process and the presence of the fluorescent dye.

**Figure 6.**
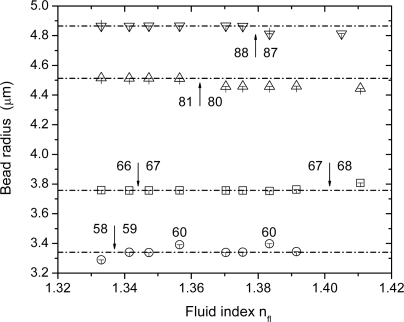
Bead radii of the different beads studied in dependence of the fluid index, n_fl_, of the DI water/glycerol mixture injected into the microfluidic flow cell as determined by the fitting procedure. The arrows indicate a step up or down of the corresponding mode numbers. The errors shown are the Δ_±10%_ errors calculated from a variation of Equation 2. For details, see main text.

Another reason for the observed size dependence of the results is related to the effects of particle dimension on the evaluation procedure. First of all, because the free spectral range scales inversely with the bead radius, fewer and fewer modes fall into the spectral range of the detection system, thus reducing the overall information content available. To this lower number of modes add their broader widths (*cf.* Figure 4), which makes determination of their positions more uncertain and thus more susceptible to an erroneous interpretation. The effect of this worse primary data condition can be nicely seen from the dependence of the calculated bead radii, R, and mode numbers, $\ell =\nu -\frac{1}{2}$, which are shown in Figure 6 in dependence of the fluid index, n_fl_. Typically, the spectrum evaluation on basis of Equation 2 is very stable with respect to the best-fit mode number, ℓ. With increasing fluid index, however, the peak positions become less and less certain and the mode numbers start to fluctuate, typically by ±1. Since the mode number defines basically, how many wavelengths fit into a bead circumference, with every change in the mode number also the best-fit bead radius undergoes an alteration. This can be seen well in Figure 6, where the arrows indicate a change in the mode number. For the largest bead under evaluation, this uncertainty sets in at a relatively high fluid index of about 1.38, for the second largest bead around 1.36, and for the smallest microbead radii studied around 1.34. Each time the mode number steps up or down, a change in radius can be observed, thus corroborating the expected correlation between ℓ and R. For the smallest bead radius, R = 3.3 μm, the mode number changes frequently, indicating that here a limit in precision is reached due to the uncertainty in determining the mode positions. Over a wide range of fluid indices, n_fl_, and bead radii, R, the determination of the environmental index of the microbeads, n_e_, seems to be unaffected from this correlation between ℓ and R (*cf.* Figure 5) and thus reveals that n_e_ and R are sufficiently decoupled to allow their simultaneous determination on basis of Equations 1 and 2.

Another cause for the deviation from linearity in the evolution of the environmental index, n_e_, with the fluid index n_fl_, could be related to the presence of the substrate and/or interfacial effects, such as an enrichment of glycerol in vicinity of the surface. An influence of the substrate on the bandwidths of WGM has already been discussed in the literature. Le Thomas *et al*. [18] calculated a maximum mode broadening of about Δλ = 3 nm for surface-adsorbed 6 μm PS beads in air (n_substrate_ = 1.5). In our case, the broadening should be even higher due to the lower index contrast, m = n_s_/n_e_, and the slightly higher substrate index of n_glass_ = 1.5255. We speculate that some of the above mentioned broad features we observe in the background of the larger beads studied (R ≈ 5 μm; *cf.* Figure 1a), may have their origin in such coupling effects between surface and WGMs. Since we described these features by additional Voigt profiles during the fitting, the undistorted information about those WGM shifts exclusively caused by index changes could be extracted from the data. As we mentioned above, this was not possible for the smaller beads studied as well as the large beads at high fluid indices, where we used only a minimum number of Voigt profiles for description of the clearly discernible modes because of their significant broadening. Therefore, we cannot exclude that in these regimes the results of the fitting routine are somewhat influenced by WGM-surface coupling as described by Le Thomas *et al*. and that this is one cause for the observed deviations from the expected behavior for bead sizes below 8 μm. This issue requires some further clarification in the future.

Also interfacial effects, such as an influence of the flow conditions or particular glycerol-surface interactions, are more likely to be experienced by smaller beads. They are, however, less likely because the SPR experiments performed on a flat surface bearing the same surface chemistry showed no deviation from linear behavior of the SPR response with the fluid index. It should also be noted that when changing the fluid in the microfluidic cell, great care was taken to remove the former DI water/glycerol mixture by rinsing the flow cell with a copious amount of DI water. Subsequently, the flow cell was flushed thoroughly with the next mixture before continuing the measurements. Therefore, poor fluid exchange cannot be the cause of the observed effects. Whatever the cause might be, interfacial effects should be independent of the way of data evaluation and therefore already be observable in the raw data. In fact, in Figure 3, for the two smallest microbeads, there is some indication of a deviation of the mode positions from the expected dependency in particular for fluid indices close to that of water. This may be an indication that some effects take place during the initial exposure of the microbeads to the DI water/glycerol mixture. Why this can be observed only with smaller beads may be either addressed to their higher sensitivity to environmental changes or to said interfacial effects. Further work will be required to elucidate these questions in more detail.

This brings us to a more general error discussion. We found that the fit results based on Equation 2 for simultaneous determination of the parameters ν, m, and R are quite stable. The error bars shown in Figures 5 and 6 correspond to the ±10% boundaries of the minimum deviation Δ_min_ found for the respective best fit, i.e., $\left(\Delta_{\pm 10\%}-\Delta_{\text{min}}\right)/\Delta_{\text{min}}\overset{!}{=}0.1$. These errors turned out to be very small, thus indicating that the evaluation procedure yields quite robust results. It should be noted that the best-fit deviation Δ, which is the sum of all deviations of 4 to 6 mode positions, is typically <0.25 nm, which means that the individual experimental modes deviate from their calculated counterparts by less than 0.042 nm. Therefore, the main errors introduced into the procedure are of experimental origin. Here, those imposed by insufficient knowledge of the exact mode positions are most crucial. It should be stressed that the number of free parameters in the algorithm is small and that with only few assumptions, e.g., by setting n_e_ = 1.333 for the environmental index in the case of a DI water environment, absolute values for n_s_, n_e_, ν, and R can be calculated for the entire parameter range studied. This implies, however, that also the mode positions need to be precisely determined on an absolute scale. It was for this reason that the spectral range of the detection system had been calibrated to a number of well-known laser lines (*cf.* experimental section), which reduces the uncertainty mainly to the reproducibility by which the mechanical stage of the monochromator can be set to a certain wavelength. The manufacturer’s test sheet of our instrument gives a turret repeatability of ±0.067 nm and a drive repeatability of ±0.002 nm, so that we can safely assume ±0.07 nm as upper limit for the error of the absolute wavelength scale. To these errors adds that of the determination of the peak centers via the fitting of Voigt profiles (*cf.* Figures 1 and 3), which depends severely on bead radius and fluid index. A safe upper limit for this uncertainty is ±0.5 nm, yielding a total error in the peak determination of ±0.6 nm.

To get a clue on how crucial this uncertainty in the mode positions affects the determination of the different parameters, we calculated the corresponding partial derivatives, $\frac{\partial X}{\partial \lambda_{\mathrm{WGM}}}$, for n_s_, n_e_, and R, via Mie-Debye theory (the exact solutions was used here to exclude any influence of the Airy approximations on the error calculation) and from those the respective maximum errors $\Delta X=\frac{\partial X}{\partial \lambda_{\mathrm{WGM}}}\;\Delta \lambda_{\mathrm{WGM}}$, where X is one of the parameters of interest and *Δλ_WGM_* = 0.6 nm. The results, which are listed in Table 2 for the smallest and the largest particle radii studied, indicate that also the experimental errors are quite small. This holds particularly for the bead index, n_s_. Its large deviation from the known bulk value of PS of Δn_s_ ≈ 0.03 can obviously not been explained by a wrong absolute determination of the wavelength scale. Also, the deviations found in n_e_ particularly for small bead radii (*cf.* Figure 5) are beyond the boundaries of the experimental errors. It should be further noted that the errors under consideration are maximum errors. For certain restrictions in particle dimension and/or fluid indices, the errors can be much smaller. Up to fluid indices of 1.36, for example, the error in the mode positions is smaller than 0.025 nm irrespective of the bead radius and smaller than 0.005 nm for all bead radii except the smallest (R = 3.3 μm). The corresponding errors in the parameters n_s_, n_e_, and R are also given in Table 2 for comparison.

## 5. Experimental Section

### 5.1. Materials

Polystyrene (PS) microspheres with nominal diameters of 6–10 μm and yellow-green fluorescent 10 μm PS microspheres were purchased from Polysciences, Inc. (Warrington, PA, USA); poly(allylamine hydrochloride) (PAH), MW ∼15,000 Da, poly(sodium 4 styrenesulfonate) (PSS), MW ∼70,000 Da, 11-mercaptohexadecanoic acid (MHA), and xylene, p.a. grade, were received from Sigma-Aldrich K. K. (Tokyo, Japan); glycerol, >99%, was obtained from Wako Pure Chemical Industr., Ltd. (Osaka, Japan), Coumarin 6 laser grade (C6G) dye from MP Biomedicals (Solon, OH, USA) and polydimethylsiloxane (Sylgard 184; PDMS) from Dow Corning Co. (Midland, MI, USA); all chemicals were used as received. Microscopy cover slips, 32 × 24 × 0.17 mm^3^, were obtained from Matsunami Glass Industr., Ltd. (Osaka, Japan); uncoated Sensor Au chips for SPR were purchased from Biacore K. K. (Tokyo, Japan) DI water was produced with a Milli-Q system from Millipore, S.A, (Molsheim, France).

### 5.2. Methods

#### Microsphere sample preparation

PS microspheres were doped with C6G using a liquid two-phase system. A saturated C6G/xylene solution was placed on top of a diluted microsphere suspension in de-ionized (DI) water and stirred until the xylene had completely evaporated. The suspension was then washed by centrifugation, removal of the supernatant and subsequent replacement of the lost volume by DI water. This rinsing step was repeated twice before coating the doped beads with two polyelectrolyte (PE) double layers of PAH/PSS following the layer-by-layer deposition method described elsewhere [22]. Microscope glass cover slips were coated with PAH/PSS layers [23] and terminated with a PAH layer in order to promote adhesion of the PSS-coated microbeads, which were deposited on the surface from highly diluted suspension via drop-coating. To ensure fixation of the beads even at high fluid viscosity, surface and deposited beads were coated with two more double layers of PAH/PSS. Then, the glass substrate was attached to a microfluidic flow cell made of PDMS bearing a rectangular flow channel of 15 × 2 × 0.1 mm^3^ in size.

#### Optical set-up

For observation and detection of the fluorescence emission of the microsphere sensors a Nikon TS100 inverted microscope was applied (Nikon Co., Tokyo, Japan). The 441.6 nm emission of a cw-HeCd laser (Kimmon Lasers, Tokyo, Japan) was used for fluorescence excitation of the particles in order to excite WGMs from their interior upon optical pumping. The beam was guided via a free-beam set-up to the top of the microscope stage and then focused from above through the PDMS flow cell onto the bead by applying a quartz lens with a focal length of +75 mm. The excitation power as measured before the focusing lens was set to 10–20 μW depending on the intensity of the selected sensor’s fluorescence emission. For the latter’s spectral analysis, a monochromator (Triax 550, 2,400L/mm grating; Horiba Jobin Yvon, Tokyo, Japan) and a cooled charge-coupled device (CCD) camera (DU440; Andor Technology, Belfast, N. Ireland) were applied. Excitation light scattered into the detection path was filtered off the fluorescence signal by means of a 2 mm Schott G475 color glass filter. Acquisition time settings of the CCD camera were 60 s per spectrum for small beads (R < 4.6 μm) and 6–15 s per spectrum for large ones. Wavelength positions of the CCD/monochromator system were calibrated by measuring a number of laser lines (HeCd at 441.60 nm, Ar Ion at 457.935 nm, 487.9864 nm, and 514.531 nm) and subsequent variance analysis.

#### In-situ sensing experiments

After mounting the fluid cell on the microscope stage, a number of microbeads (typically 5–6) inside of the microfluidic channel were selected and measured in air to check on the quality of their WGM spectra. Then, DI water/glycerol mixtures of increasing glycerol content were subsequently injected into the flow cell by means of a glass syringe. In-between, i.e., before injecting the next mixture, the former one was removed by purging the flow cell with a copius amount (∼3 mL) of DI water. For each fluid, the selected beads were measured under the respective conditions. For DI water/glycerol mixtures beyond 70% glycerol content the fluid became too viscous for injection into the microfluidic system. At the end of each experiment, the microfluidic cell was once more filled with DI water to check on permanent changes of the WGM spectra of the measured beads. This experimental sequence was performed four times and a total of 19 beads were studied, from which four representatives were selected for a detailed analysis of their WGM spectra as given above.

#### Determination of refractive indices

The refractive indices of the different DI water/glycerol mixtures were calculated by means of the relation given by Foley *et al*. [19] To account for minor deviations from the wanted mixing ratio, i.e., refractive index, the mixtures were subsequently injected into a surface plasmon resonance (SPR) system (Biacore X, Biacore K.K., Tokyo, Japan), and the corresponding change in resonance units plotted as a function of the theoretical values. The plot was linearly fitted and the fit results used for correction of the calculated index values. The SPR gold chips used for these measurements were first functionalized with MHA (from 2mM ethanolic solution after 1 hour UV-ozone treatment of the respective gold chip), then 2 double layers of PAH/PSS were deposited in-situ on top of the MHA to yield the same outer surface coating as that of the microbeads.

## 6. Conclusions

Summarizing, the present study reveals that the Airy approximations for description of WGM positions can be successfully implemented into a fitting routine for simultaneous determination of all relevant parameters, i.e., mode number, $\ell =\nu -\frac{1}{2}$, refractive index contrast, m, and microbead radius, R, on an absolute scale. With only one assumption for the environmental index, n_e_, under known conditions, e.g., for a microbead immersed into pure water, the bead index, n_s_, can be determined and exploited for sensing of unknown environments with a resolution of up to Δn_e_ = ±(2.1 ± 0.26) 10^−4^. By means of this approach we found that with n_s_ ≈ 1.56, the bead index is significantly smaller than expected from studies on similar, however, non-dyed PS microbeads [21]. Application of this experimentally determined bead index allows precise tracing of environmental indices down to a sensor diameter of about 8 μm despite of the presence of the surface and the PE coating used for bead fixation. Below this size, some deviations occur that are most likely caused by the presence of the surface and some interfacial effects in its vicinity.

What lessons we can learn from these results in view of in-situ sensing applications? For refractive index sensing, for example, it is important to know that for surface-adhered beads the influence of the substrate can be neglected and thus enables sensing even in small structures, such as microfluidic devices. For biosensing, on the other hand, it is interesting that the environmental index of the bulk solution can be determined independently from the presence of a surface functionalization as required, for example, to mediate specific binding to wanted analytes. The reason for this particularity is related to another observation made here: our results show that in the evaluation scheme applied bead radius and environmental index are sufficiently decoupled from each other to allow their independent determination. It seems therefore that a thin surface coating, such as the PE layers used for bead fixation on surface, alters mainly the overall bead size, but has only little influence on the determination of the environmental index, n_e_. This can be easily understood when envisioning that the evanescent field, on whose scale the index is sensed by the microbead, extents into the bulk fluid by more than 100 nm [11], while the thickness of the PE coating amounts only to few nanometers [22]. On the other hand, in prior work the feasibility of thin film sensing was already demonstrated [3,4], so that the robustness of the method with respect to the determination of the fluid index must be seen as an advantage, not as a lack of sensitivity. The direct proof of this concept, however, will be the target of a future study.

As mentioned, the sensitivity of detecting adsorption layers on the microbead surface scales inversely with its radius, thus suggesting its minimization for adlayer sensing applications, such as biosensing. Our work shows that for sensing in an aqueous environment, this size is limited to about 8 μm below which deviations from the expected behavior occur. Somewhat unfortunate for biosensing, which is typically performed in buffer media with fluid indices not to different from that of water, i.e., typically n_fl_ < 1.36, the observed deviation is most pronounced in the regime of n_fl_ = 1.33 – 1.36. In their recent work, Meissner and coworkers studied the applicability of latex microspheres with a nominal radius of R = 5 μm decorated with quantum dots to refractive index sensing in exactly this refractive index regime (n_fl_ = 1.33 – 1.36) and found a sensitivity, i.e., WGM shift, five times that of the theoretical prediction [2]. The authors explain this enhancement with the presence of the quantum dots, which may drastically raise the refractive index of the outer bead volume. In our case, the increase occurs only at smaller bead dimension and to lesser extent (about 1.6 times the expected behavior). It is interesting, however, that it can be observed in the same index regime and that an increased bead index can obviously be not the reason. Therefore, whether these similarities in the findings are accidental or the effect of a common underlying cause needs to be clarified in future studies.

Altogether, our work demonstrates that optical sensing on the microscale by means of WGM excitations in fluorescent microbeads may be rendered into a highly precise and easily applicable analytical tool, which is expected to be of high interest for a variety of fields and thus may find ample application in the future.

## Figures and Tables

**Table 1.** Calculated bead indices, n_s_, as a function of bead radii for surface-adsorbed and freely floating beads.

**Surface-Adsorbed**		**Freely Floating**	

Radius, R (μm)	Bead index, n_s_	Radius, R (μm)	Bead index, n_s_
3.3	1.5530	5.0	1.5615
3.8	1.5573	5.4	1.5645
4.5	1.5609	5.5	1.5700
4.9	1.5623	5.5	1.5592

Mean	1.5584 ± 0.0042		1.5638 ± 0.0047

**Table 2.** Experimental errors for bead index, n_s_, environmental index, n_e_, and bead radius, R, in dependence of the uncertainty in determining the mode positions, Δλ, for the smallest and largest bead studied.

	**R = 3.3 μm**		**R = 4.9 μm**	

	**TM**	**TE**	**TM**	**TE**

	**Δλ = 0.6 nm**			
Δn_s_	±0.0085	±0.0112	±0.0146	±0.0189
− Δn_e_	±0.0023	±0.0022	±0.0021	±0.0020
ΔR	±3.881	±3.889	±5.695	±5.700

	**Δλ = 0.025 nm (n_fl_ < 1.36)**			

Δn_s_	±0.00036	±0.00047	±0.00061	±0.00079
− Δn_e_	±9.47E-05	±8.96E-05	±8.64E-05	±8.45E-05
ΔR	±0.1617	±0.1621	±0.2373	±0.2375

	**Δλ = 0.005 nm (n_fl_ < 1.36, R > 3.3 μm)**			

Δn_s_	±7.1E-05	±9.35E-05	±0.00012	±0.00016
− Δn_e_	±1.89E-05	±1.79E-05	±1.73E-05	±1.69E-05
ΔR	±0.0323	±0.0324	±0.0475	±0.0475
